# Response to Letter from Wong on determining the target difference in sample size calculations for randomised controlled trials

**DOI:** 10.1186/s13063-024-08023-x

**Published:** 2024-03-02

**Authors:** Jonathan A. Cook, Richard A. Parker

**Affiliations:** 1 Centre for Statistics in Medicine, Nuffield Department of Orthopaedics, Rheumatology and Musculoskeletal Sciences, University of Oxford, Oxford, UK; 2 Edinburgh Clinical Trials Unit, Usher Institute, University of Edinburgh, Edinburgh, UK

We would like to thank Wong for their response to our article on the relevance of clinical importance when determining the target difference in sample size calculations and clarification on their position [1, 2]. It is with the hope of making clearer the differences in our positions that we make this further response, though we note we do agree on a number of practical points such as the challenges in estimation and the impact of the trial context [1]. As before, we consider the setting of designing a phase III or “definite” randomised controlled trial (RCT). The target difference refers to the treatment effect specified in the sample size calculation conducted when designing the study to detect a difference between treatments (i.e. a superiority contrast). We refer to Wong’s proposal as the “true benefit” approach to specification of the target difference in the sample size calculation, based on our understanding of the method [1, 3]. Unless otherwise stated, we assume a conventional (frequentist) Neyman-Pearson approach is being implemented which is currently the most common sample size determination approach used for RCTs.

We reaffirm our view that the *importance* of the target difference value is relevant and should be considered when determining the sample size for a phase III RCT [2]. To clarify, we have *not* argued that the target difference should *always* be the minimum important difference (MID). It may be appropriate for the target difference to be a value larger than the MID in some settings. Indeed, sometimes the benefits in terms of reduced sample size, shorter study duration, or reduced patient burden, would outweigh the risks of being underpowered to detect a difference as small as the MID [4]. Alternatively, a larger difference than the MID might be considered necessary given other considerations related to the use of a treatment (e.g. adverse events). Consider for example, the interesting psilocybin example put forward by Wong [1]. Wong found that the sample size calculation was highly sensitive to the value of the target difference and that the trial became “infeasible” when the target difference was set to be the MID [1]. Wong also commented that values above the MID were perceived to be more realistic based on prior data [1]. In this case then, we agree that setting a value of the target difference that is higher than the MID should be considered. Where we differ with Wong, is that we still think the MID should be a consideration in choosing a suitable value for the target difference. In other words, we should not ignore what we think would be an important difference completely when determining the required study sample size. In practice, this might mean setting a value for the target difference some way in-between the MID and that which is deemed to be most “plausible” or realistic based upon previous evidence. By doing so we have not ignored the MID, rather we have used it as a marker to help determine an appropriate target difference. Our approach is relatively modest in that we think importance of the proposed target difference is a relevant consideration when specifying the target difference in the sample size calculation, as well as when deciding whether to conduct the study. The main benefit of setting the target difference to the MID, if one were to do so, is that it ensures that the study has the required statistical power across a range of possible differences that are viewed as “important” to one or more stakeholder groups, assuming of course, that all the other assumptions of the sample size calculation are appropriate.

Nevertheless, we think that Wong touches on a helpful point that we should be careful in sample size calculations that we do not ignore the study context or the sensitivity of our calculations to the inputs we have used [1]. Indeed, we think that rigidly adhering to the MID for a given outcome for all trials, regardless of the study context or sensitivity of the sample size calculations, would not constitute good research practice. For ease of discussion, we do not consider uncertainty related to the magnitude of an important difference or what would constitute the MID here, though we readily acknowledge that this will affect specification of a sample size calculation and interpretation of findings once the study has been completed [5]. Similarly, we will refer to a sample size “calculation” here though we acknowledge some RCT designs will require simulation-based approaches as opposed to a direct calculation to determine a suitable sample size. In this case, the underlying issue remains the same even though the complexity of the process of determining an appropriate sample size may be greater.

It is perhaps useful to emphasise that statistical power is a planning concept; it relates to a hypothetical situation relevant to the study of interest and provides reassurance against sampling variability. One way to think of this is to imagine conducting thousands of studies with the same sample size and analysing each of them individually. Power is the proportion of these studies which would detect the effect of interest (as specified in the sample size calculation e.g. 2-sided *p*-value ≤ 0.05) if it really exists. It is relevant to note that commonly the calculation performed in a sample size calculation for a RCT is a simplification of the planned analysis. In particular, the sample size calculation is typically based on a single primary outcome, whereas in reality a number of study outcomes were collected. Furthermore, it is often not reasonable to assume with confidence relevant nuisance parameters which have a bearing on the estimation of the treatment effect (e.g. the correlation between multiple baseline factors and the outcome of interest) [6]. Therefore, the stated power is often in practice an educated approximation (aside from considerations of the specification of the target difference).

The problematic nature of the “true benefit” approach, in our view, relates to the requirement that the “assumed benefit must match the true benefit”[1] for the sample size is be “valid”, and that a “valid” sample size calculation must “reflect truth about what the trial will accomplish in the real word”[1]. In our view no sample size calculation for a difference in the treatment effect can ever bear this burden. In the presence of variability, we can never guarantee what the analysis will show: only how likely or not it is to show something (e.g. a non-zero mean difference at the 2-sided 5% significance level) under various assumptions, and commonly under a simplified scenario. The true difference could in fact be zero, i.e. there is no “true benefit”. In that case, there is no “valid” sample size for a test of superiority under this way of thinking, which is in our view an incoherent premise. It might be countered that we will never do a trial to detect no difference and will always expect some effect, but we may be wrong about that.

As we have argued previously, we conduct RCTs in order to assess whether there is any benefit of a treatment or intervention [2]. By conducting a phase III RCT we acknowledge we are unsure what the “true benefit” is, and even accept the possibility of a detrimental effect (or at least no effect). So why would we make the design of such studies dependent upon the magnitude of the estimate of the quantity of which we are unsure enough about to conduct a new study? It is impossible to be sure if the power was “true” or not as Wong defines it and as he acknowledges. Wong substitutes the estimate from prior evidence for the true effect. However, that may be or may not be a sensible decision depending on the context and the reliability of the prior evidence. The unspecified presumption in Wong argument is that the existing evidence about what the relevant effect is, at least roughly, at the right level. On the contrary, the literature is replete with examples of RCTs which have overturned the prior evidence. See Rothwell and Hall for example for some specific examples [7–10]. Djulbegovic and colleagues, based upon a review of 860 RCTs, suggest that around 50% of RCTs comparing a new treatment to a standard treatment demonstrate the new treatment is better than the standard one [9]. In statistical parlance the “true benefit” approach assumes the estimates from previous studies are unbiased and precise. This is in our view a heroic assumption to apply widely. If the desire is to incorporate the existing evidence into the analysis alongside the data from the new study (i.e. the RCT) there are various ways to do this but that is a different concern, and a different strategy from the “true benefit” approach. The “true” treatment effect might be viewed as that which applies to a given study with the relevant population, treatments, outcome, and analysis. In other words, the observed treatment effect will be conditional on all of these factors (as well as sampling errors etc.). So unless the previous studies upon which the sample size is being based have been conducted in a consistent manner as the planned RCT, the estimate will not correspond to (exactly) the same treatment effect, even on average. Indeed, pilot studies or phase II studies are often based on a select, and generally sicker, population compared to the clinical population that a phase III trial recruits from.

In our view, the true benefit approach would tend to lead in practice to trials that do not detect true clinically relevant treatment differences as the estimates of treatment effect based upon prior published evidence will generally be inflated. Prior evidence tends to suggest larger effects due to publication bias or selection bias, which dissipate when a large phase III RCT is conducted [7–9]. The approach advocated by Wong might also lead to an RCT not being conducted on the basis of misleading estimate of the “true benefit” from studies of dubious quality. It is true that under our approach, we could still be wrong about the treatment effect, and the difference could be zero or the difference could be much higher than expected, but in either case, our sample size calculation provides coverage *if* the true difference was “important” as long as we have selected the target difference accordingly (and the other inputs are not far off). The point of a sample size calculation is to cover a range of possibilities, the vast majority of which will not transpire in any single study. We need to ensure that a sample size calculation is robust to different values of the treatment difference, preferably ensuring it addresses at least some scenarios of interest.

Given that we argue that the derivation of sample size should *not exclusively* be based on the likelihood of observed effect sizes from previous evidence for a phase III trial, we would argue that there is no necessity to explicitly incorporate uncertainty about the underlying treatment effect into sample size calculations (unless we wish to incorporate uncertainty about what would be of important to us). If one wishes to incorporate prior evidence into the estimation of the treatment effect then a Bayesian approach would be the natural one to adopt, though not strictly necessary. This, however, is a separate issue per se, from what we would wish to detect, which is still a consideration. Consideration of both what may be realistic given prior evidence and what we wish to detect is relevant in our view for a phase III trial. Bayesian conceptions of sample size determination in the past have perhaps not always given enough thought to what it is desirable to be able to detect as opposed to detecting the likely effect given what we currently know. This though is by no means true of all such approaches (e.g. Bayesian indifference zones [11]). Kunzmann et al. suggest a “prior-quantile approach” to utilising the MID as the target difference, by assessing “power” in “a Bayesian way” via a quantile of the prior distribution (as opposed to the mean as per common practice) [4]. We agree that this is a useful approach for specifying the target difference and ensuring suitable operating criteria. However, as we have argued above, we are sceptical as to how often one would have reasonable knowledge on the true difference, and so specification of an appropriate prior distribution in order to use this method may be challenging.

The approach we have argued for can be readily implemented in the conventional (Newman-Pearson, frequentist) paradigm; it is not necessary to introduce Bayesian considerations unless the planned analysis of a study is Bayesian. In the context of sample size derivations based on conditioning on *hypothetical* values for the true underlying difference, we would argue that it is perfectly natural that such values would be assumed to be fixed from the perspective of the researcher*.* Therefore, we continue to advocate careful consideration of the importance of the target difference regardless of whether the proposed framework is frequentist or Bayesian.

## Data Availability

Not applicable.
